# γ-Secretase Components as Predictors of Breast Cancer Outcome

**DOI:** 10.1371/journal.pone.0079249

**Published:** 2013-11-01

**Authors:** Hanna M. Peltonen, Annakaisa Haapasalo, Mikko Hiltunen, Vesa Kataja, Veli-Matti Kosma, Arto Mannermaa

**Affiliations:** 1 Institute of Clinical Medicine, Pathology and Forensic Medicine, University of Eastern Finland, Kuopio, Finland; 2 Biocenter Kuopio and Cancer Center of Eastern Finland, University of Eastern Finland, Kuopio, Finland; 3 Imaging Center, Clinical Pathology, Kuopio University Hospital, Kuopio, Finland; 4 Institute of Clinical Medicine – Neurology, University of Eastern Finland, Kuopio, Finland; 5 Institute of Clinical Medicine, Oncology, University of Eastern Finland, Kuopio, Finland; 6 Cancer Center, Kuopio University Hospital, Kuopio, Finland; Health Canada and University of Ottawa, Canada

## Abstract

γ-secretase is a large ubiquitously expressed protease complex composed of four core subunits: presenilin, Aph1, PEN-2, and nicastrin. The function of γ-secretase in the cells is to proteolytically cleave various proteins within their transmembrane domains. Presenilin and Aph1 occur as alternative variants belonging to mutually exclusive γ-secretase complexes and providing the complexes with heterogeneous biochemical and physiological properties. γ-secretase is proposed to have a role in the development and progression of cancer and γ-secretase inhibitors are intensively studied for their probable anti-tumor effects in various types of cancer models. Here, we for the first time determined mRNA expression levels of presenilin-1, presenilin-2, Aph1a, Aph1b, PEN-2, and nicastrin in a set of breast cancer tissue samples (N = 55) by quantitative real-time PCR in order to clarify the clinical significance of the expression of different γ-secretase complex components in breast cancer. We found a high positive correlation between the subunit expression levels implying a common regulation of transcription. Our univariate Kaplan-Meier survival analyses established low expression level of γ-secretase complex as a risk factor for breast cancer specific mortality. The tumors expressing low levels of γ-secretase complex were characterized by high histopathological tumor grade, low or no expression of estrogen and progesterone receptors and consequently high probability to fall into the class of triple negative breast cancer tumors. These results may provide novel tools to further categorize breast cancer tumors, especially the highly aggressive and poorly treatable breast cancer type of triple negative cases, and suggest a significant role for γ-secretase in breast cancer.

## Introduction

γ-secretase is a large ubiquitously expressed protease complex composed of four core subunits: presenilin (PS), Aph1, PEN-2, and nicastrin (NCT). These subunits are necessary and sufficient for the protease activity of γ-secretase [1,2]. γ-secretase cleaves various type I membrane proteins by regulated intramembrane proteolysis [1,3]. The γ-secretase-mediated cleavage releases the C-terminal intracellular domain (ICD) of the substrate protein which may then execute important signaling functions inside the cell. The group of the γ-secretase substrates is large and constantly growing encompassing already more than 90 members [3]. Many of the identified substrates are intimately involved in tumorigenesis. Examples of these proteins include Notch receptors and their ligands, CD44, ErbB4, E-cadherin, and MUC1. γ-secretase may influence on tumorigenesis also via its role in angiogenesis as many of the γ-secretase substrates (e.g. Notch, VEGFR-1, IGF1R, ErbB4, cadherins, and APP) are shown to regulate the formation and development of new blood vessels [4]. Thus γ-secretase inhibitors are intensively studied for their anti-tumor effects in various types of cancer models [2,5,6]. Several reports have described inhibitory effects of these compounds on breast cancer cell growth via down-regulation of Notch signaling pathway which is aberrantly activated in breast cancer [6-8]. While previous studies have described the effects of γ-secretase inhibitors on cancer cells especially concentrating on only one of the γ-secretase substrates at the time (for example Notch or E-cadherin), the multiplicity of γ-secretase substrates suggests that the observed effects can be mediated via the inhibited cleavage of multiple substrates and subsequently altered signaling pathways. In addition to abnormal expression and function of many substrate proteins, the expression and/or activity of γ-secretase complex itself can be disturbed during tumorigenesis.

γ-secretase subunits presenilin and Aph1 occur as alternative variants: PS1/PS2 and Aph1a/Aph1b [1,3]. Furthermore Aph1a can be alternatively spliced to short or long splice variant: Aph1aS or Aph1aL [9,10]. These variants seem to be differentially expressed among mouse, rat and human tissues [11-16] and to belong to mutually exclusive γ-secretase complexes [9,10,17-19]. Consistently, many studies have suggested distinct yet overlapping biochemical and physiological roles for the subunit isoforms [11,13,20-29]. Altogether at least six distinct γ-secretase complexes with different subunit composition and with varying enzymatic activities and physiological outcomes can be formed. It is highly possible that perturbations in the equilibrium of γ-secretase complex components leading to profound effects on enzyme activity underlie some physiological disturbances. For example, a shift from the predominance of complexes containing PS1 and/or Aph1a towards a greater proportion of γ-secretase complexes containing PS2 and/or Aph1b could be one factor leading to the development of Alzheimer’s disease [26,28]. We hypothesized that a similar unbalance in the presence of distinct γ-secretase complexes might be associated with the development and progression of breast cancer. Thus we wanted to clarify the clinical significance of the expression of γ-secretase components in breast cancer. We aimed to resolve whether one of the distinct γ-secretase complex types is preferentially expressed in breast cancer and whether the expression levels of different γ-secretase components are associated with tumorigenesis, histopathological subtypes of the tumor, or breast cancer outcome. Here, we report a strong positive correlation between the mRNA expression levels of the γ-secretase subunits PS1, PS2, Aph1a, Aph1b, PEN-2, and NCT indicating a tight co-regulation of the transcription. We are able to establish low level of γ-secretase complex as a risk factor for breast cancer specific mortality and to reveal the association of low level of γ-secretase complex with triple negative type of breast cancer.

## Materials and Methods

### Patients

Fresh frozen tissue samples from 55 breast cancer cases (54 in the case of NCT) in the Kuopio Breast Cancer Project [30-33] were used in this study. Table 1 summarizes the clinicopathological data of the cases. The mean age at the time of breast cancer diagnosis was 62.6 years and all the study subjects were female. The patients were followed up until death or November 2009. The Kuopio Breast Cancer Project has been approved by the joint ethics committee of Kuopio University and Kuopio University Hospital (written consents 1/1989 and 61/2010). Each patient gave informed written consent for participation in the study.

**Table 1:** The clinicopathological data of the patients (N = 55).

**Variable**	**N (%)**
**Patients age (years)**	
≤ 51.9	17 (30.9)
≥ 52	38 (69.1)
**Histopathological grade**	
1	8 (14.5)
2	28 (50.9)
3	19 (34.5)
**Stage**	
1	12 (21.8)
2	42 (76.4)
3	0 (0.0)
4	1 (1.8)
**Tumor type**	
ductal	40 (72.7)
lobular	9 (16.4)
other	6 (10.9)
**Estrogen receptor**	
negative	14 (25.5)
positive	41 (74.5)
**Progesterone receptor**	
negative	22 (40.0)
positive	33 (60.0)
**Her2 receptor**	
0-2	50 (90.9)
3	4 (7.3)
No data	1 (1.8)
**Triple negativity**	
ER=0 / PR=0 /Her2=0-2	10 (18.2)
positive	45 (81.8)
**Patient status**	
Dead, breast cancer	20 (36.4)
Dead, other cause	21 (38.2)
Alive, no recurrence	11 (20.0)
Alive, recurrence	3 (5.5.)
**Mean follow-up time (days)**	3371.2 [38-6713]

### RNA extraction, cDNA preparation, and quantitative real-time RT-PCR

Tissue specimens were stored at -70°C. RNA extraction and cDNA preparation were done essentially as described by Nykopp et al. (2009) [34]. TaqMan® Gene Expression Assays (Applied Biosystems) for the genes studied were Hs00997789_m1 (PS1), Hs01577197_m1 (PS2), Hs00211268_m1 (Aph1a), Hs00229911_m1 (Aph1b), Hs01033961_g1 (PEN-2), Hs00950933_m1 (NCT), and Hs99999904_m1 (PPIA), which was used as the endogenous control [35]. Standard curves were established by cDNA obtained by reverse transcription of 2 μg of Human Breast Total RNA (Ambion®). Each sample and each point of the standard curve was performed in triplicate reactions. The maximum deviation between the expression values of each triplicate sample was allowed to be 0.3. The mean value of the triplicates was used as the raw expression value. Relative gene expression values were calculated as the ratio between the target gene and the endogenous control (cyclophilin A, PPIA) and were used in the statistical analyses. 

### *In silico* databases of human transcriptomes

The GeneSapiens database (http://www.genesapiens.org) was used to analyse previously published results of the gene expression levels of γ-secretase subunits in human breast cancer [36]. The database contains 1,504 different human breast carcinoma samples from publicly available Affymetrix microarray experiments. In order to study gene expression levels of γ-secretase subunits in triple negative subtype of breast cancer, mRNA expression (Agilent microarray) data of The Cancer Genome Atlas (TCGA, http://cancergenome.nih.gov) were downloaded from the cBioPortal for Cancer Genomics (http://www.cbioportal.org) [37,38]. The selected TCGA dataset [39] contains 81 basal-like breast tumors and 445 tumors of other subtypes. 80 % of basal-like tumors were characterized as triple negative breast cancers. 

### Statistical methods

Statistical analyses were carried out using SPSS Statistics 17.0 for Windows (SPSS Inc.). P ≤ 0.05 was considered significant in all analyses. Correlation between the expression levels of γ-secretase subunits determined in this study were analysed by the Spearman’s non-parametric test of correlation. When comparing the expression levels of single γ-secretase subunits with known clinicopathological characteristics of the tumors (Table 1), differences between groups were analysed by non-parametric Mann-Whitney U-test in the case of two groups and by non-parametric Kruskal-Wallis test when multiple groups were included in the same comparison. Binary variables of individual γ-secretase subunits were created by dividing the samples in two groups based on the mean of the relative gene expression values of the specific subunit (Table S1). The expression values below the mean were designated as 0 and above the mean as 1. Binary variables were used to describe the division of low and high expressing samples between various sample groups and in univariate survival analyses with the Kaplan-Meier method and log-rank test. Breast cancer survival was defined as the time between the date of diagnosis and the date of death due to breast cancer. Deaths by other causes were censored. The descriptive values (sample size, mean, and standard deviation) of low and high expressing sample groups are presented in Table S1. A common variable (named γ-secretase) to describe the overall expression level of γ-secretase complex in the samples was created by summarizing the zeros and ones of the binary variables of individual subunits. This variable with six ranks was used to calculate mean values, standard deviations and p-values presented in Table 2. Further, a binary γ-secretase variable was established by dividing the variable into low (ranks 0, 1, and 2) and high (ranks 3, 4, 5, and 6) expressing sample groups and used in Table 2 and in Kaplan-Meier analysis. 

**Table 2:** Association of mRNA expression level of γ-secretase complex with clinicopathological characteristics of the tumors.

	**γ-secretase**			
**Variable**	Low (%)	High (%)	Mean ± SD^a^	P-value^b^
**Histopathological grade**				
1	4 (14.3)	4 (15.4)	3.25 ± 2.32	0.136
2	13 (46.4)	14 (53.8)	2.74 ± 1.95	
3	11 (39.3)	8 (30.8)	1.79 ± 1.87	
**Estrogen receptor**				
negative	12 (42.9)	2 (7.7)	0.93 ± 1.33	<0.001**
positive	16 (57.1)	24 (92.3)	3.03 ± 1.94	
**Progesterone receptor**				
negative	16 (57.1)	6 (23.1)	1.55 ± 1.77	0.004**
positive	12 (42.9)	20 (76.9)	3.13 ± 1.95	
**Her2 receptor**				
0-2	25 (89.3)	24 (96.0)	2.59 ± 2.04	0.144
3	3 (10.7)	1 (4.0)	1.00 ± 1.41	
**Triple negativity**				
yes	8 (28.6)	2 (7.7)	1.00 ± 1.49	0.006**
no	20 (71.4)	24 (92.3)	2.82 ± 1.98	

a Mean and standard deviation of γ-secretase complex expression values of the samples belonging to each separate sample group

b P-values of γ-secretase variable with six ranks by non-parametric Mann-Whitney U-test (or by non-parametric Kruskal-Wallis test in the case of histopathological grade)

** Association is significant at the 0.01 level

## Results

### Expression levels of γ-secretase subunits have a significant mutual correlation

mRNAs of γ-secretase subunits PS1, PS2, Aph1a, Aph1b, PEN-2, and NCT were analysed by real-time quantitative PCR utilizing TaqMan® technology. Gene expression levels varied considerably between the samples. We found no preference for any of the subunit variants (PS1/PS2 and Aph1a/Aph1b) over the other in our sample set of breast cancer tissues. Instead, a significant positive correlation between the expression levels of γ-secretase subunits was observed (Table 3). In order to analyse previously published data on the gene expression of γ-secretase subunits in human breast cancer, we used the GeneSapiens *in silico* database of human transcriptomes [36]. The analysis revealed a similar significant positive correlation between the expression levels of γ-secretase subunits (Table S2) as observed in our sample set.

**Table 3:** Correlation between mRNA expression levels of γ-secretase subunits PS1, PS2, Aph1a, Aph1b, PEN-2, and NCT determined by Spearman’s non-parametric correlation test.

	**PS2**	**Aph1a**	**Aph1b**	**PEN-2**	**NCT**
**PS1**	0.726**	0.759**	0.737**	0.150	0,576**
**PS2**		0.709**	0.692**	0.237	0.671**
**Aph1a**			0.695**	0.343*	0.577**
**Aph1b**				0.388**	0.569**
**PEN-2**					0.350**

* Correlation is significant at the 0.05 level

** Correlation is significant at the 0.01 level

### Expression of γ-secretase subunits associates with tumor grade and hormone receptor status

We compared the expression levels of single γ-secretase subunits with known clinicopathological characteristics of the tumors (Table 1). The main findings are presented in Tables S3, S4, S5, S6, S7 and S8. The mRNA expression of all γ-secretase subunits was lower in the sample group with tumor grade 3 than in lower grade tumors. The association between subunit expression and tumor grade was significant for Aph1b (p = 0.033; Table S6), PEN-2 (p = 0.005; Table S7), and NCT subunits (p = 0.043; Table S8). We found a significant association between mRNA expression of γ-secretase subunits and protein expression of the estrogen (ER) and progesterone (PR) hormonal receptors. Low expression of γ-secretase subunits was associated with low expression of the receptors. Aph1b (p = 0.032; Table S6) and PEN-2 subunits (p = 0.005; Table S7) had a significant association with human epidermal growth factor receptor 2 (Her2), but in this case the expression level of the subunits was lower in the sample group with high Her2 protein expression than in other tumors. 

### Triple negative breast cancer subtype is characterized by low expression of γ-secretase subunits

We compared the expression levels of γ-secretase subunits in the sample group of triple negative breast cancer cases (N = 10) to the expression in cancer tissues expressing at least one of the receptors ER, PR, or Her2 and found a significant association with PS2 (p = 0.002; Table S4), Aph1a (p = 0.038; Table S5), Aph1b (p = 0.002; Table S6), and PEN-2 subunits (p = 0.025; Table S7). Interestingly, the expression levels of γ-secretase subunits were lower in triple negative breast cancer cases than in other samples. In order to validate this finding in larger sample set, we utilized publicly available TCGA dataset [39] containing mRNA expression data of 526 breast cancer tumor samples. The expression levels of PS1, PS2, and Aph1b were significantly lower in the sample group of basal-like tumors (N = 81) than in tumors of other subtypes (N = 445; p < 0,001; Table S9). 

### Expression of γ-secretase subunits associates with clinical outcome

**Figure 1:**
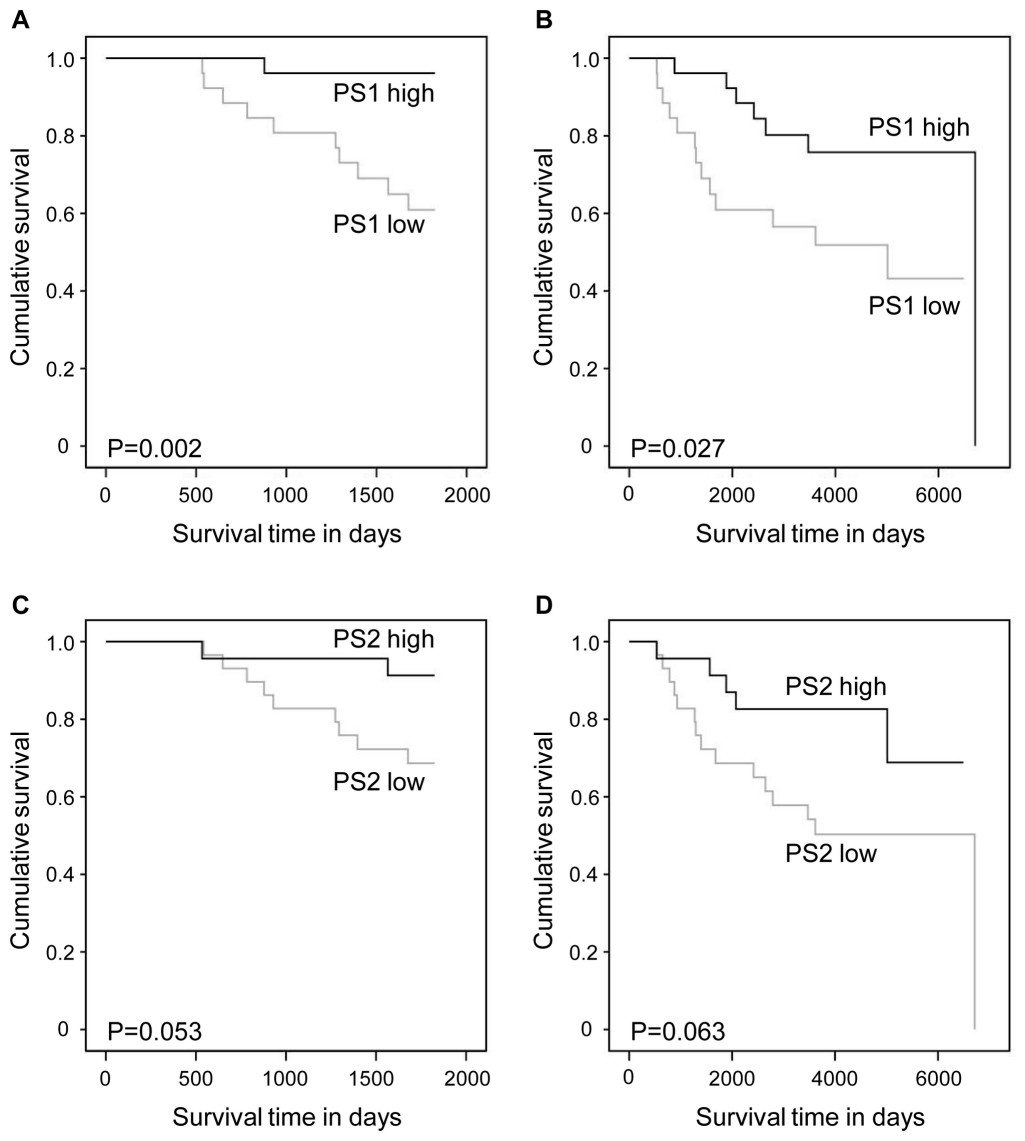
Breast cancer specific survival in Kaplan-Meier univariate analysis according to the expression levels of presenilins. Low (N = 28) and high (N = 27) mRNA levels of PS1 (A and B) and low (N = 32) and high (N = 23) mRNA levels of PS2 (C and D) at 5 years (A and C) and the whole follow-up time (B and D).

**Figure 2:**
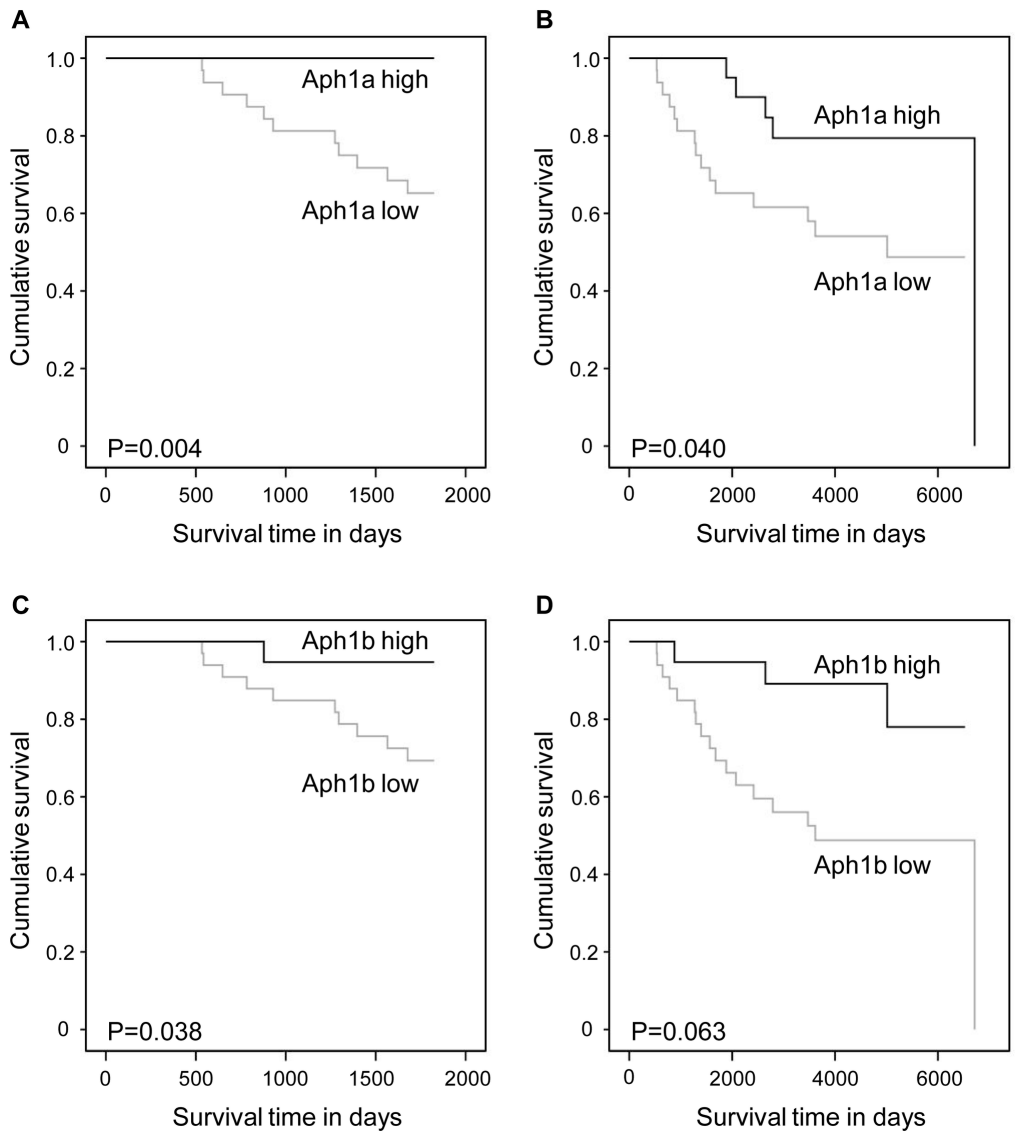
Breast cancer specific survival in Kaplan-Meier univariate analysis according to the expression levels of Aph1 variants. Low (N = 34) and high (N = 21) mRNA levels of Aph1a (A and B) and low (N = 34) and high (N = 21) mRNA levels of Aph1b (C and D) at 5 years (A and C) and the whole follow-up time (B and D).

**Figure 3:**
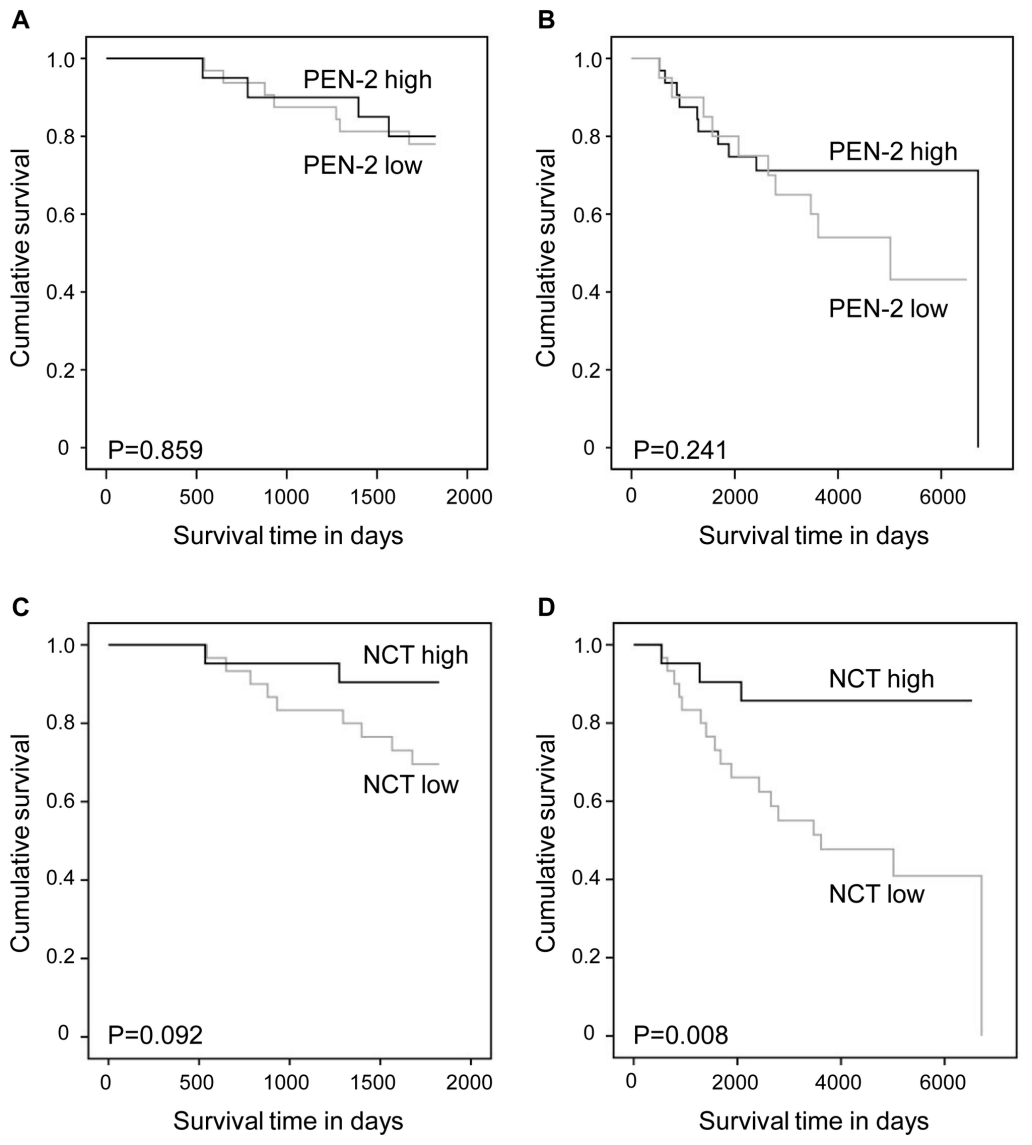
Breast cancer specific survival in Kaplan-Meier univariate analysis according to the expression levels of PEN-2 and nicastrin. Low (N = 33) and high (N = 22) mRNA levels of PEN-2 (A and B) and low (N = 33) and high (N = 21) mRNA expression levels of NCT (C and D) at 5 years (A and C) and the whole follow-up time (B and D).

We performed Kaplan-Meier survival analyses in order to detect the possible prognostic role of the expression of γ-secretase subunits in breast cancer specific survival. For the analysis, we divided the samples into low and high expressing groups based on the mean of the relative gene expression values of the specific subunit. The descriptive values (sample size, mean, and standard deviation) of these groups are presented in Table S1. The minimum and maximum follow-up times were 38 days and over 18 years, respectively (Table 1). In addition to follow-up survival, also the 5 year survival was established. The survival curves are presented in Figures 1–3. There was a significant association between the low expression levels of PS1, Aph1a, Aph1b, and NCT and poor breast cancer specific survival. 

### γ-secretase ensemble has clinical significance

Because of the significant positive correlation of the expression levels of γ-secretase subunits, we created one common variable (named γ-secretase) to describe the overall expression level of γ-secretase complex in the samples (see Materials and Methods for details). The values of γ-secretase variable were compared with known clinicopathological characteristics of the tumors (Table 1). The results of the comparisons (Table 2) reinforce our findings with individual subunits showing a strong association of γ-secretase complex with ER and PR and a decreased expression of the enzyme complex in triple negative breast cancer cases. 

**Figure 4:**
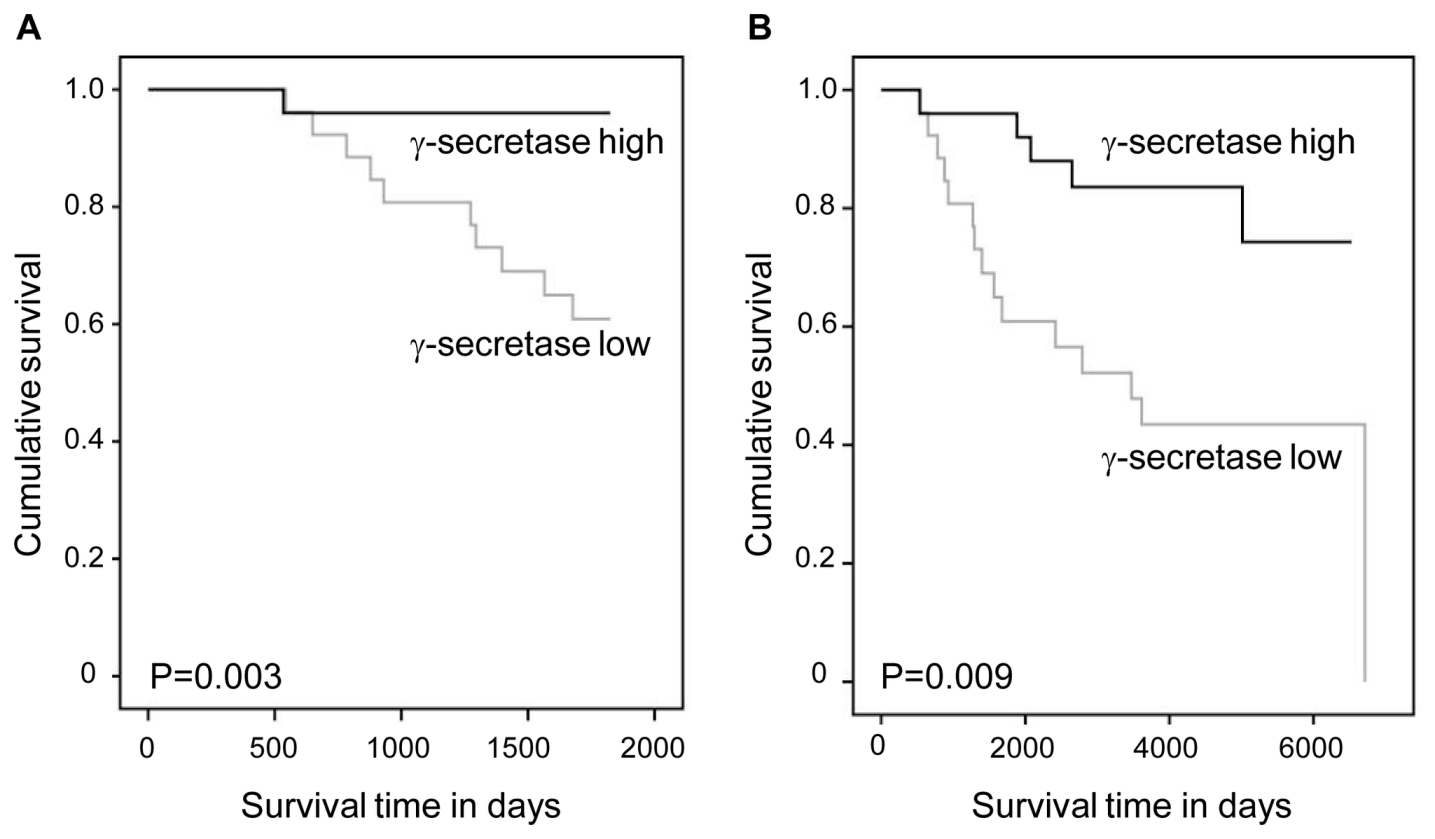
Breast cancer specific survival in Kaplan-Meier univariate analysis according to the expression of γ-secretase complex. Low (N = 28) and high (N = 26) expression levels of γ-secretase complex at 5 years (A) and the whole follow-up time (B).

The possible involvement of γ-secretase complex in the breast cancer specific survival was examined by Kaplan-Meier survival analysis (Figure 4). Low expression of γ-secretase complex predicted significantly poorer survival than the higher expression levels.

γ-secretase subunit variants PS1/PS2 and Aph1a/Aph1b are suggested to belong to mutually exclusive γ-secretase complexes [9,10,17-19]. Thus with the subunit variants explored in this study, four distinct γ-secretase complexes can be formed. Therefore we created four novel variables to describe the overall expression level of each complex type. This was performed substantially with the same procedure which was used to create γ-secretase variable but excluding the subunit variants not involved in a specific complex type. Complex type variables showed mutual significant positive correlations (data not shown). Further analyses of the novel variables resulted in highly similar outcomes as the ones of γ-secretase variable (data not shown).

### Exceptionally low or high level of γ-secretase complex characterizes specific tumor subtypes

Finally, we wanted to more closely study the characteristics of the tumors with very low (rank 0) or high (rank 6) expression of the γ-secretase complex (Table 4). There were 13 cases with the rank 0 (low expression of all the individual subunits of γ-secretase) in γ-secretase variable. The majority of these tumors (62 %) were classified to have a grade 3. The expression of ER was significantly lower in these tumors than in other cases (p = 0.002). 62 % of the tumors did not express ER. There were significantly more triple negative breast cancer cases in this subgroup of the samples than in other samples (p = 0.008). In Kaplan-Meier survival analysis, low level of γ-secretase complex was associated with poor prognosis. The association was statistically significant at 5 years (p = 0.040). 

**Table 4:** The characteristics of the tumors expressing exceptionally low (rank 0) or high (rank 6) levels of γ-secretase complex.

**γ-secretase**	**N**	**Histopathological grade**	**ER**	**PR**	**HER2**	**Triple negativity**
0	13	3	-*	-	+	yes*
6	4	1/2	+	+	-	no

* designates a statistically significant difference determined by non-parametric Mann-Whitney U-test

The 4 samples with high expression of γ-secretase complex (rank 6 in γ-secretase variable) were characterized by exceptionally low histopathological grade, high expression of ER and PR, and low expression of Her2. None of the cases was of grade 3 or triple negative or expressed high levels of Her2, but all of them expressed ER. Because of the small number of highly expressing samples, it was not possible to show any significance in statistical tests for differences between the groups defined by specific clinicopathological characteristics of the tumors or in Kalpan-Meier survival analysis. All the 4 patients with tumors expressing high levels of γ-secretase subunits were still alive or had died because of other causes than breast cancer in the end of the follow-up time. 

## Discussion

The potential role of γ-secretase in the development and progression of cancer has been widely accepted [5,40]. To date, most studies have concentrated on investigating the expression levels and function of γ-secretase substrates [41-48] or the effects of γ-secretase inhibitors in breast cancer [49-53]. Limited attention has been given to investigating the expression and function of individual γ-secretase components. To our knowledge this is the first report describing mRNA expression levels of γ-secretase subunits PS1, PS2, Aph1a, Aph1b, PEN-2, and NCT in breast cancer tissues and establishing low level of γ-secretase complex as a risk factor for breast cancer specific mortality. Our data provides novel tools to characterize and categorize the diversity of breast cancer tumors. Of special importance is our finding of the association of low expression level of γ-secretase complex with triple negative type of breast cancer. 

In this study, we first aimed to investigate the mRNA expression levels of γ-secretase subunits in human breast cancer specimens in order to observe possible predominance of some subunit variants over the others in this cancer type. Our results demonstrate a strong positive correlation between the mRNA expression levels of the γ-secretase subunits PS1, PS2, Aph1a, Aph1b, PEN-2, and NCT (Table 3) indicating a tight co-regulation of the expression of these transcripts. *In silico* transcriptome analysis utilizing the GeneSapiens database gave further support to our observation. This finding was unexpected in the light of previous studies showing differential expression of PS1/PS2 and Aph1a/Aph1b among various tissues [11,12,14-16] and the compensatory expression of other members of PS or Aph1 families when the endogenous expression of their counterparts have been artificially suppressed [10,23,29]. However, our results are in line with the studies showing joint expression levels for the various γ-secretase components [12,18,54]. It seems that all four proteins closely regulate each other. Knocking out or over-expressing one of the γ-secretase subunits decreases and increases the expression levels of other components, respectively [16,18,21,25,27,29,55-57]. Previous studies have been conducted at protein level and the regulation mechanism has been suggested to involve stabilization, maturation, or degradation of the proteins. Our results at mRNA level indicate that also the level of transcription is tightly co-regulated. It is possible that γ-secretase complex controls the transcription of its own subunits via a feedback loop [58,59].

Next we wanted to untangle whether there was association between the expression levels of γ-secretase subunits and the clinicopathological characteristics of the tumors in breast cancer (Tables S3, S4, S5, S6, S7 and S8). We obtained very similar results with all the tested γ-secretase components (PS1, PS2, Aph1a, Aph1b, PEN-2, and NCT) and with the common γ-secretase variable (Table 2) which we used to describe the expression level of the whole complex. This was not surprising as the expression levels of the individual subunits closely followed those of each other. Filipovic et al. (2011) studied protein expression of NCT and found significant association with histopathological tumor grade and with hormonal receptor (ER and PR) expressions [60]. Our results are in a full agreement with those findings: The mRNA expression of Aph1b, PEN-2, and NCT were significantly lower in breast cancer cases with high tumor grade and there was a significant association between high expression level of γ-secretase complex and hormonal receptors. Previous research has established a cross-talk between Notch and ER in breast cancer [61-63]. Activation of Notch or inhibition of γ-secretase has differential effects on ER negative and positive breast cancer cells [61]. Estradiol acting on ER inhibits Notch and amyloid-β precursor protein signaling [63,64]. We can only speculate whether the cross-talk of Notch and ER is mediated via γ-secretase activity. One tempting explanation for the observed high γ-secretase complex level in ER expressing tumors is that down-regulation of Notch signaling in ER-positive cells induces a compensatory effect increasing the expression of γ-secretase complex. Triple negative breast cancer type is characterized by the lack of expression of ER and PR. Furthermore Her2 is not over-expressed. These tumors tend to be high grade and the disease is aggressive with high recurrence, metastatic, and mortality rates [65]. Hormonal receptor and Her2 antagonists are ineffective in the treatment of this breast cancer type and therefore there is an urgent need for better therapeutics for this form of cancer [65]. Previous studies have demonstrated a heavy dependence of triple negative breast cancers on Notch signaling and suggested γ-secretase inhibitors as effective drugs for this breast cancer type [61-63,66,67]. Unfortunately our study does not give clear support to the idea of utilizing γ-secretase inhibitors to treat triple negative breast cancer as the expression of γ-secretase subunits was significantly lower in this breast cancer type than in the other cases. Our *in silico* analysis of publicly available TCGA dataset [39] supported this finding (Table S9). However, it is highly possible that already a small amount of γ-secretase complexes exhibits significant activity and that the low level of γ-secretase complex expression observed in triple negative breast cancer tumors is sufficient to produce elevated levels of activated Notch species in the conditions of high expression of Notch receptors and ligands typical for triple negative tumors [62,68,69]. Our further studies with larger sample set of triple negative breast cancer tissue samples will elucidate this matter.

Our results indicate that the tumors expressing low levels of γ-secretase complex are characterized by higher histopathological tumor grade, low or no expression of hormonal receptors and consequently higher probability to fall into the class of triple negative tumors (Table 4). They seem to be more aggressive and poorly treatable and probably more fatal. Thus it was reasonable to investigate whether the components of γ-secretase complex had prognostic value in breast cancer. Kaplan-Meier survival analysis revealed that low expression level of γ-secretase complex was associated with poor breast cancer specific survival. Interestingly, the same trend was also observed with triple negative breast cancer cases (N = 10) only (data not shown), although the effect of the expression of γ-secretase complex was not significant. The finding was unexpected from the point of view of multiple previous studies suggesting γ-secretase inhibitors alone or in combination with other therapeutics as efficient drugs for breast cancer [6-8]. The principal rationale behind this therapeutic intervention is the aberrant Notch signaling in breast cancer, which leads to increased proliferation, restricted differentiation, impaired apoptosis and enhanced maintenance of putative cancer stem cells [43,52,70,71]. However, multiple lines of evidence suggest that the effect of γ-secretase or Notch inhibition on cancer cells is far from straightforward. The cytotoxicity of γ-secretase inhibitors to breast cancer cells might not be mediated via inhibition of Notch signaling but via proteasome inhibition [72]. Notch signaling itself is highly context-dependent and there is some evidence that Notch homologues may have opposite effects in breast cancer [73,74]. Although Notch deregulation appears to have oncogenic effects in numerous solid tumors, important exceptions exist. Notch-1 has been shown to play an important tumor-suppressive role in epidermal keratinocytes [75-77] and studies in cervical, prostate, lung, brain and liver cancers have also suggested tumor-suppressive function for Notch signaling [78]. It is possible that both the tumor-suppressive and oncogenic properties of Notch are taking place at the same time, and the final outcome is dependent on the cellular context [78,79]. Accordingly, Notch signaling may have multiple divergent roles also in breast cancer cells. If we further consider γ-secretase with dozens of identified substrates besides Notch receptors [3] and possible hundreds of downstream targets, it becomes evident that the enzyme may play multiple and even opposite roles in the development and progression of cancer. Underlining this notion, γ-secretase has been reported to function as a tumor suppressor in epithelia via Notch signaling as well as via epidermal growth factor receptor and β-catenin [80-85]. In our further studies, we will aim to untangle the main pathways utilized by γ-secretase in breast cancer and the possible correlation between the expression of γ-secretase complex and the activity of these pathways.

Overall, a tempting explanation for many of our results may be a feedback mechanism used to compensate the potentially reduced activity of γ-secretase or Notch by increasing transcription of γ-secretase subunits. In this case, already a small level of γ-secretase complexes would achieve required level of activity in the cells. The increased expression levels, on the other hand, would reflect reduced activity. This idea receives some support from a recent study on the biological activity of γ-secretase inhibitor PF-03084014 in breast cancer xenograft models. The inhibitor was shown to induce significant tumor growth inhibition, robust impairment of Notch signaling, and significant upregulation of the mRNA expression level of NCT in HCC1599 model [86]. It has to be kept in mind that only a small percentage of PS is engaged in catalytically active complexes [24,87] and the same is probably true for the other subunits as well. Thus it is indeed highly possible that the expression of a certain subunit protein does not reflect the amount of active complex in the cells. This leads to the conclusion that the expression levels of γ-secretase subunits observed in this study might not result in the increased expression at protein level and eventually in the increased activity of the enzymatic complex. However, many previous studies have described altered mRNA expression with a direct consequence of aberrant enzymatic activity [25,55,60,88-91]. 

All the interesting results introduced here were achieved by a sample set of 55 breast cancer tissues. This number of samples was clearly sufficient for the present study giving firm answers to our research problems. We obtained highly similar results with all of the γ-secretase subunits tested and with our γ-secretase variable which greatly increases the reliability of the results. Our *in silico* analyses gave further support to the findings. The results implicate an independent additional effect of low mRNA expression of γ-secretase complex along with the other known risk factors on breast cancer specific survival. However, further studies will naturally benefit from larger sample size. As we cannot completely exclude the possibility of unspecific effects and synergy of other tumor characteristics (ER, PR, tumor grade) having an effect on our survival results, further studies using multivariate survival analyses with larger sample groups are needed to clarify the independence of the γ-secretase effect. 

In conclusion, this is to our knowledge the first report describing mRNA expression levels of γ-secretase subunits PS1, PS2, Aph1a, Aph1b, PEN-2, and NCT in breast cancer tissues. We demonstrated a high positive correlation between the expression levels of γ-secretase subunits implying a common regulation of transcription. We discerned a firm association of γ-secretase with ER and PR, a finding nicely in line with previous results obtained studying expression of NCT in breast cancer [60] and certainly deserving further investigation. We designated γ-secretase complex expression as a potential tool to categorize breast cancer tumors: Tumors with low γ-secretase complex expression typically lack hormone receptors and have a poor prognosis based on higher histopathological tumor grade and lower breast cancer specific survival. Furthermore, we show the association between γ-secretase complex expression and triple negativity of the breast cancer cases. These findings thus pave the way for exploring the role of the γ-secretase complex in triple negative breast cancer and for further categorizing this severe cancer type.

## Supporting Information

Table S1 **Sample size, mean and standard deviation of the relative gene expression values of γ-secretase subunits PS1, PS2, Aph1a, Aph1b, PEN-2 and NCT (All) and the same descriptive values of the low and high expressing sample groups categorized based on the mean above (Low and High).**
(DOCX)

Table S2 **Correlation between mRNA expression levels of γ-secretase subunits PS1, PS2, Aph1a, Aph1b, PEN-2, and NCT using the GeneSapiens *in silico* database (http://www.genesapiens.org) in human breast carcinoma samples (N = 757 - 953).**
(DOCX)

Table S3 **Association of mRNA expression of presenilin 1 (PS1) with clinicopathological characteristics of the tumors.**
(DOCX)

Table S4 **Association of mRNA expression of presenilin 2 (PS2) with clinicopathological characteristics of the tumors.**
(DOCX)

Table S5 **Association of mRNA expression of Aph1a with clinicopathological characteristics of the tumors.**
(DOCX)

Table S6 **Association of mRNA expression of Aph1b with clinicopathological characteristics of the tumors.**
(DOCX)

Table S7 **Association of mRNA expression of PEN-2 with clinicopathological characteristics of the tumors.**
(DOCX)

Table S8 **Association of mRNA expression of nicastrin (NCT) with clinicopathological characteristics of the tumors.**
(DOCX)

Table S9 **Association of mRNA expression of γ-secretase subunits PS1, PS2, Aph1a, Aph1b, PEN-2 and NCT with basal-like disease subtype in publicly available TCGA (http://cancergenome.nih.gov) dataset of 526 breast cancer tumor samples [39].**
(DOCX)
