# Supplementary material for: γ-Secretase Components as Predictors of Breast Cancer Outcome
Source: PLoS One. 2013 Nov 1;8(11):e79249. doi: 10.1371/journal.pone.0079249 (PMC3815159; doi:10.1371/journal.pone.0079249)
Supplement: Table S1 — Sample size, mean and standard deviation of the relative gene expression values of γ-secretase subunits PS1, PS2, Aph1a, Aph1b, PEN-2 and NCT (All) and the same descriptive values of the low and high expressing sample groups categorized based on the mean above (Low and High). (DOCX) [file pone.0079249.s001.docx]

| **Variable** | **N (%)** | **Mean ± SD** |
| --- | --- | --- |
| **PSEN1** |  |  |
| All | 55 | 0.49  ± 0.44 |
| Low | 28 (50.9) | 0.27 ± 0.09 |
| High | 27 (49.1) | 0.72 ± 0.23 |
| **PSEN2** |  |  |
| All | 55 | 1.55 ± 1.15 |
| Low | 32 (58.2) | 0.83 ± 0.32 |
| High | 23 (41.8) | 2,56 ± 0.76 |
| **Aph1a** |  |  |
| All | 55 | 0.97± 0.85 |
| Low | 34 (61.8) | 0.62 ± 0.21 |
| High | 21 (38.2) | 1.53 ± 0.41 |
| **Aph1b** |  |  |
| All | 55 | 0.53 ± 0.38 |
| Low | 34 (61.8) | 0.27 ± 0.12 |
| High | 21 (38.2) | 0.96 ± 0.48 |
| **PEN-2** |  |  |
| All | 55 | 1.11 ± 0.96 |
| Low | 33 (60.0) | 0.67 ± 0.24 |
| High | 22 (40.0) | 1.77 ± 0.44 |
| **NCT** |  |  |
| All | 54 | 0.54 ±0.47 |
| Low | 33 (61.1) | 0.36 ± 0.10 |
| High | 21 (38.9) | 0.82 ± 0.21 |
