# Supplementary material for: γ-Secretase Components as Predictors of Breast Cancer Outcome
Source: PLoS One. 2013 Nov 1;8(11):e79249. doi: 10.1371/journal.pone.0079249 (PMC3815159; doi:10.1371/journal.pone.0079249)
Supplement: Table S2 — Correlation between mRNA expression levels of γ-secretase subunits PS1, PS2, Aph1a, Aph1b, PEN-2, and NCT using the GeneSapiens in silico database (http://www.genesapiens.org) in human breast carcinoma samples (N = 757 - 953). (DOCX) [file pone.0079249.s002.docx]

|  | **PS2** | **Aph1a** | **Aph1b** | **PEN-2** | **NCT** |
| --- | --- | --- | --- | --- | --- |
| **PS1** | 0.13** | 0.068 | 0.16** | 0.39** | 0.18** |
| **PS2** |  | 0.18** | 0.25** | 0.22** | 0.18** |
| **Aph1a** |  |  | 0.22** | 0.34** | 0.37** |
| **Aph1b** |  |  |  | 0.012 | 0.079* |
| **PEN-2** |  |  |  |  | 0.22** |

* Correlation is significant at the 0.05 level

** Correlation is significant at the 0.01 level
