# Supplementary material for: γ-Secretase Components as Predictors of Breast Cancer Outcome
Source: PLoS One. 2013 Nov 1;8(11):e79249. doi: 10.1371/journal.pone.0079249 (PMC3815159; doi:10.1371/journal.pone.0079249)
Supplement: Table S3 — Association of mRNA expression of presenilin 1 (PS1) with clinicopathological characteristics of the tumors. (DOCX) [file pone.0079249.s003.docx]

|  | **PS1** | | | |
| --- | --- | --- | --- | --- |
| **Variable** | Low (%) | High (%) | Mean ± SD^a^ | P-value^b^ |
| **Histopathological grade** |  |  |  |  |
| 1 | 4 (14.3) | 4 (14.8) | 0.52 ± 0.34 | 0.52 |
| 2 | 13 (46.4) | 15 (55.6) | 0.53 ± 0.30 |  |
| 3 | 11 (39.3) | 8 (29.6) | 0.42 ± 0.23 |  |
| **Estrogen receptor** |  |  |  |  |
| negative | 10 (35.7) | 4 (14.8) | 0.36 ± 0.22 | 0.029* |
| positive | 18 (64.3) | 23 (85.2) | 0.53 ± 0.29 |  |
| **Progesterone receptor** |  |  |  |  |
| negative | 15 (53.6) | 7 (25.9) | 0.39 ± 0.23 | 0.033* |
| positive | 13 (46.4) | 20 (74.1) | 0.55 ± 0.30 |  |
| **Her2 receptor** |  |  |  |  |
| 0-2 | 24 (88.9) | 26 (96.3) | 0.50 ± 0.29 | 0.2 |
| 3 | 3 (11.1) | 1 (3.7) | 0.32 ± 0.21 |  |
| **Triple negativity** |  |  |  |  |
| yes | 7 (25.0) | 3 (11.1) | 0.37 ± 0.24 | 0.105 |
| no | 21 (75.0) | 24 (88.9) | 0.51 ± 0.29 |  |

^a^ Mean and standard deviation of PS1 expression values of the samples belonging to each separate sample group

^b^ P-values of relative gene expression of PS1 by non-parametric Mann-Whitney U-test (or by non-parametric Kruskal-Wallis test in the case of histopathological grade)

* Association is significant at the 0.05 level
