# Supplementary material for: γ-Secretase Components as Predictors of Breast Cancer Outcome
Source: PLoS One. 2013 Nov 1;8(11):e79249. doi: 10.1371/journal.pone.0079249 (PMC3815159; doi:10.1371/journal.pone.0079249)
Supplement: Table S4 — Association of mRNA expression of presenilin 2 (PS2) with clinicopathological characteristics of the tumors. (DOCX) [file pone.0079249.s004.docx]

|  | **PS2** | | | |
| --- | --- | --- | --- | --- |
| **Variable** | Low (%) | High (%) | Mean ± SD^a^ | P-value^b^ |
| **Histopathological grade** |  |  |  |  |
| 1 | 4 (12.5) | 4 (17.4) | 1.71 ± 1.10 | 0.401 |
| 2 | 16 (50.0) | 12 (52.2) | 1.67 ± 1.10 |  |
| 3 | 12 (37.5) | 7 (30.4) | 1.31 ± 0.85 |  |
| **Estrogen receptor** |  |  |  |  |
| negative | 14 (43.8) | 0 (0.0) | 0.75 ± 0.36 | <0.001** |
| positive | 18 (56.3) | 23 (100) | 1.83 ± 1.03 |  |
| **Progesterone receptor** |  |  |  |  |
| negative | 18 (56.3) | 4 (17.4) | 1.09 ± 0.82 | 0.002** |
| positive | 14 (43.8) | 19 (82.6) | 1.86 ± 1.03 |  |
| **Her2 receptor** |  |  |  |  |
| 0-2 | 29 (90.6) | 21 (95.5) | 1.59 ± 1.03 | 0.188 |
| 3 | 3 (9.4) | 1 (4.5) | 1.10 ± 1.02 |  |
| **Triple negativity** |  |  |  |  |
| yes | 10 (31.3) | 0 (0.0) | 0.77 ± 0.41 | 0.002** |
| no | 22 (68.8) | 23 (100.0) | 1.73 ± 1.04 |  |

^a^ Mean and standard deviation of PS2 expression values of the samples belonging to each separate sample group

^b^ P-values of relative gene expression of PS2 by non-parametric Mann-Whitney U-test (or by non-parametric Kruskal-Wallis test in the case of histopathological grade)

** Association is significant at the 0.01 level
