# Supplementary material for: γ-Secretase Components as Predictors of Breast Cancer Outcome
Source: PLoS One. 2013 Nov 1;8(11):e79249. doi: 10.1371/journal.pone.0079249 (PMC3815159; doi:10.1371/journal.pone.0079249)
Supplement: Table S5 — Association of mRNA expression of Aph1a with clinicopathological characteristics of the tumors. (DOCX) [file pone.0079249.s005.docx]

|  | **Aph1a** | | | |
| --- | --- | --- | --- | --- |
| **Variable** | Low (%) | High (%) | Mean ± SD^a^ | P-value^b^ |
| **Histopathological grade** |  |  |  |  |
| 1 | 3 (8.8) | 5 (23.8) | 1.19 ± 0.64 | 0.244 |
| 2 | 18 (52.9) | 10 (47.6) | 1.01 ± 0.60 |  |
| 3 | 3 (38.2) | 6 (28.6) | 0.80 ± 0.34 |  |
| **Estrogen receptor** |  |  |  |  |
| negative | 12 (35.3) | 2 (9.5) | 0.69 ± 0.31 | 0.016* |
| positive | 22 (64.8) | 19 (90.5) | 1.06 ± 0.57 |  |
| **Progesterone receptor** |  |  |  |  |
| negative | 17 (50.0) | 5 (23.8) | 0.80 ± 0.43 | 0.051 |
| positive | 17 (50.0) | 16 (76.2) | 1.08 ± 0.58 |  |
| **Her2 receptor** |  |  |  |  |
| 0-2 | 30 (90.9) | 20 (95.2) | 0.99 ± 0.55 | 0.225 |
| 3 | 3 (9.1) | 1 (4.8) | 0.67 ± 0.29 |  |
| **Triple negativity** |  |  |  |  |
| yes | 9 (26.5) | 1 (4.8) | 0.66 ± 0.26 | 0.038* |
| no | 25 (73.5) | 20 (95.2) | 1.03 ± 0.56 |  |

^a^ Mean and standard deviation of Aph1a expression values of the samples belonging to each separate sample group

^b^ P-values of relative gene expression of Aph1a by non-parametric Mann-Whitney U-test (or by non-parametric Kruskal-Wallis test in the case of histopathological grade)

* Association is significant at the 0.05 level
