# Supplementary material for: γ-Secretase Components as Predictors of Breast Cancer Outcome
Source: PLoS One. 2013 Nov 1;8(11):e79249. doi: 10.1371/journal.pone.0079249 (PMC3815159; doi:10.1371/journal.pone.0079249)
Supplement: Table S6 — Association of mRNA expression of Aph1b with clinicopathological characteristics of the tumors. (DOCX) [file pone.0079249.s006.docx]

|  | **Aph1b** | | | |
| --- | --- | --- | --- | --- |
| **Variable** | Low (%) | High (%) | Mean ± SD^a^ | P-value^b^ |
| **Histopathological grade** |  |  |  |  |
| 1 | 4 (11.8) | 4 (19.0) | 0.73 ± 0.49 | 0.033* |
| 2 | 16 (47.1) | 12 (57.1) | 0.56 ± 0.43 |  |
| 3 | 14 (41.2) | 5 (23.8) | 0.41 ± 0.47 |  |
| **Estrogen receptor** |  |  |  |  |
| negative | 12 (35.3) | 2 (9.5) | 0.21 ± 0.16 | <0.001** |
| positive | 22 (64.8) | 19 (90.5) | 0.64 ± 0.47 |  |
| **Progesterone receptor** |  |  |  |  |
| negative | 17 (50.0) | 5 (23.8) | 0.33 ± 0.22 | 0.005** |
| positive | 17 (50.0) | 16 (76.2) | 0.67 ± 0.52 |  |
| **Her2 receptor** |  |  |  |  |
| 0-2 | 29 (87.9) | 21 (100.0) | 0.57 ± 0.46 | 0.032* |
| 3 | 4 (12.1) | 0 (0.0) | 0.19 ± 0.17 |  |
| **Triple negativity** |  |  |  |  |
| yes | 8 (23.5) | 2 (9.5) | 0.24 ± 0.18 | 0.002** |
| no | 26 (76.5) | 19 (90.5) | 0.60 ± 0.47 |  |

^a^ Mean and standard deviation of Aph1b expression values of the samples belonging to each separate sample group

^b^ P-values of relative gene expression of Aph1b by non-parametric Mann-Whitney U-test (or by non-parametric Kruskal-Wallis test in the case of histopathological grade)

* Association is significant at the 0.05 level

** Association is significant at the 0.01 level
