# Supplementary material for: γ-Secretase Components as Predictors of Breast Cancer Outcome
Source: PLoS One. 2013 Nov 1;8(11):e79249. doi: 10.1371/journal.pone.0079249 (PMC3815159; doi:10.1371/journal.pone.0079249)
Supplement: Table S7 — Association of mRNA expression of PEN-2 with clinicopathological characteristics of the tumors. (DOCX) [file pone.0079249.s007.docx]

|  | **PEN-2** | | | |
| --- | --- | --- | --- | --- |
| **Variable** | Low (%) | High (%) | Mean ± SD^a^ | P-value^b^ |
| **Histopathological grade** |  |  |  |  |
| 1 | 3 (9.1) | 5 (22.7) | 1.40 ± 0.42 | 0.005** |
| 2 | 14 (42.4) | 14 (63.6) | 1.26 ± 0.72 |  |
| 3 | 16 (48.5) | 3 (13.6) | 0.77 ± 0.44 |  |
| **Estrogen receptor** |  |  |  |  |
| negative | 12 (36.4) | 2 (9.1) | 0.65 ± 0.35 | <0.001** |
| positive | 21 (63.6) | 20 (90.9) | 1.27 ± 0.64 |  |
| **Progesterone receptor** |  |  |  |  |
| negative | 15 (45.5) | 7 (31.8) | 0.89 ± 0.51 | 0.018* |
| positive | 18 (54.5) | 15 (68.2) | 1.26 ± 0.68 |  |
| **Her2 receptor** |  |  |  |  |
| 0-2 | 29 (87.9) | 21 (100.0) | 1.12 ± 0.56 | 0.005** |
| 3 | 4 (12.1) | 0 (0.0) | 0.46 ± 0.08 |  |
| **Triple negativity** |  |  |  |  |
| yes | 8 (24.2) | 2 (9.1) | 0.73 ± 0.38 | 0.025* |
| no | 25 (75.8) | 20 (90.9) | 1.19 ± 0.66 |  |

^a^ Mean and standard deviation of PEN-2 expression values of the samples belonging to each separate sample group

^b^ P-values of relative gene expression of PEN-2 by non-parametric Mann-Whitney U-test (or by non-parametric Kruskal-Wallis test in the case of histopathological grade)

* Association is significant at the 0.05 level

** Association is significant at the 0.01 level
