# Supplementary material for: γ-Secretase Components as Predictors of Breast Cancer Outcome
Source: PLoS One. 2013 Nov 1;8(11):e79249. doi: 10.1371/journal.pone.0079249 (PMC3815159; doi:10.1371/journal.pone.0079249)
Supplement: Table S8 — Association of mRNA expression of nicastrin (NCT) with clinicopathological characteristics of the tumors. (DOCX) [file pone.0079249.s008.docx]

|  | **NCT** | | | |
| --- | --- | --- | --- | --- |
| **Variable** | Low (%) | High (%) | Mean ± SD^a^ | P-value^b^ |
| **Histopathological grade** |  |  |  |  |
| 1 | 4 (12.1) | 4 (19.0) | 0.72 ± 0.36 | 0.043* |
| 2 | 15 (45.5) | 12 (57.1) | 0.57 ± 0.27 |  |
| 3 | 14 (42.4) | 5 (23.8) | 0.41 ± 0.16 |  |
| **Estrogen receptor** |  |  |  |  |
| negative | 11 (33.3) | 3 (14.3) | 0.39 ± 0.17 | 0.014* |
| positive | 22 (66.7) | 18 (85.7) | 0.59 ± 0.28 |  |
| **Progesterone receptor** |  |  |  |  |
| negative | 16 (48.5) | 6 (28.6) | 0.44 ± 0.18 | 0.053 |
| positive | 17 (51.5) | 15 (71.4) | 0.61 ± 0.30 |  |
| **Her2 receptor** |  |  |  |  |
| 0-2 | 30 (90.9) | 19 (95.0) | 0.55 ± 0.28 | 0.158 |
| 3 | 3 (9.1) | 1 (5.0) | 0.35 ± 0.19 |  |
| **Triple negativity** |  |  |  |  |
| yes | 8 (24.2) | 2 (9.5) | 0.42 ± 0.17 | 0.188 |
| no | 25 (75.8) | 19 (90.5) | 0.56 ± 0.28 |  |

^a^ Mean and standard deviation of PEN-2 expression values of the samples belonging to each separate sample group

^b^ P-values of relative gene expression of NCT by non-parametric Mann-Whitney U-test (or by non-parametric Kruskal-Wallis test in the case of histopathological grade)

* Association is significant at the 0.05 level
