# Supplementary material for: γ-Secretase Components as Predictors of Breast Cancer Outcome
Source: PLoS One. 2013 Nov 1;8(11):e79249. doi: 10.1371/journal.pone.0079249 (PMC3815159; doi:10.1371/journal.pone.0079249)
Supplement: Table S9 — Association of mRNA expression of γ-secretase subunits PS1, PS2, Aph1a, Aph1b, PEN-2 and NCT with basal-like disease subtype in publicly available TCGA (http://cancergenome.nih.gov) dataset of 526 breast cancer tumor samples [39]. (DOCX) [file pone.0079249.s009.docx]

|  | **Basal-like (N = 81)** | **Other (N = 445)** |  |
| --- | --- | --- | --- |
|  | Mean ± SD | Mean ± SD | P-value^a^ |
| **PSEN1** | 0.14 ± 0.44 | 0.63 ± 0.51 | <0.001** |
| **PSEN2** | 0.80 ± 0.61 | 1.17 ± 0.53 | <0.001** |
| **Aph1a** | 0.08 ± 0.78 | -0.06 ± 0.81 | 0.103 |
| **Aph1b** | 0.44 ± 0.68 | 1.31 ± 0.62 | <0.001** |
| **PEN-2** | 0.64 ± 0.52 | 0.69 ± 0.46 | 0.486 |
| **NCT** | 1.06 ± 0.58 | 0.99 ± 0.29 | 0.286 |

^a^ non-parametric Mann-Whitney U-test

** Association is significant at the 0.01 level
